# Metal Forming Tool Monitoring Based on a 3D Measuring Endoscope Using CAD Assisted Registration

**DOI:** 10.3390/s19092084

**Published:** 2019-05-05

**Authors:** Lennart Hinz, Markus Kästner, Eduard Reithmeier

**Affiliations:** Insitute of Measurement and Automatic Control, Leibniz University Hannover, Nienburger Straße 17, 30167 Hannover, Germany; markus.kaestner@imr.uni-hannover.de (M.K.); eduard.reithmeier@imr.uni-hannover.de (E.R.)

**Keywords:** endoscopy, maintenance, fringe projection, registration, metrology

## Abstract

In order to provide timely, reliable, and comprehensive data for the maintenance of highly stressed geometries in sheet-bulk metal forming tools, this article features a possible setup by combining a 3D measuring endoscope with a two-stage kinematic. The measurement principle is based on the projection of structured light, allowing time-effective measurements of larger areas. To obtain data of proper quality, several hundred measurements are performed which then have to be registered and finally merged into one single point cloud. Factors such as heavy, unwieldy specimens affecting precise alignment. The rotational axes are therefore possibly misaligned and the kinematics and the hand-eye transformation remain uncalibrated. By the use of computer-aided design (CAD) data, registration can be improved, allowing a detailed examination of local features like gear geometries while reducing the sensitivity to detect shape deviations.

## 1. Introduction

In the context of increasing automation and digitalization of integrated industrial manufacturing processes, monitoring and automated quality assurance are essential factors, strengthening the importance of new measurement approaches. Optical measurement techniques are contactless and can meet current and future emerging requirements like significantly reduced measuring times while increasing the resolution of the data [1,2].

Sheet-bulk metal forming is a newly emerging forming process, producing complex geometries due to a combination of deep drawing and bulk metal forming [3]. Possible areas of application are given by gearing or carrier elements for transmissions in the automotive industry [4]. The tools of sheet-bulk metal forming plants are stressed during operation. A challenge for maintenance service is to evaluate the condition of the tool without having accurate information about possible deviations. A quantitative assessment of possible damage is therefore mostly unfeasible. By using endoscopic devices, geometries can be imaged which would not be assessable for classical measuring devices. Additionally, recent research strives to extend the capabilities of endoscopic inspection devices from 2D to true 3D imaging [5]. This article shall give an overview of a possible forming tool inspection setup, combining a two-stage kinematic with an endoscopic measuring device, based on the fringe projection approach. By using a structured light projection and a camera-based observation, object geometries can be measured within seconds or below with a higher resolution than e.g., sensors based on the time-of-flight approach [6].

## 2. In Situ and Offline Inspection of Forming Tools

**Figure 1:**
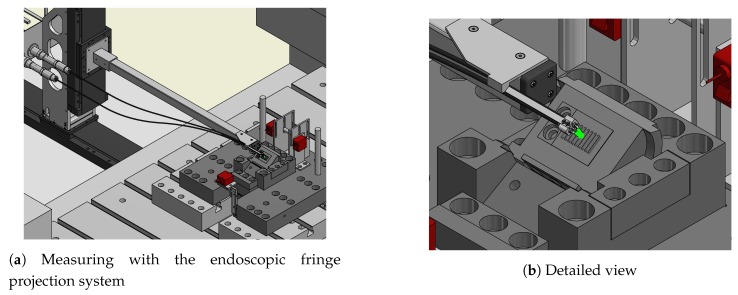
Overview of a possible in-situ measurement inside a metal forming plant (the top elements are hidden for a better perspective).

**Figure 2:**
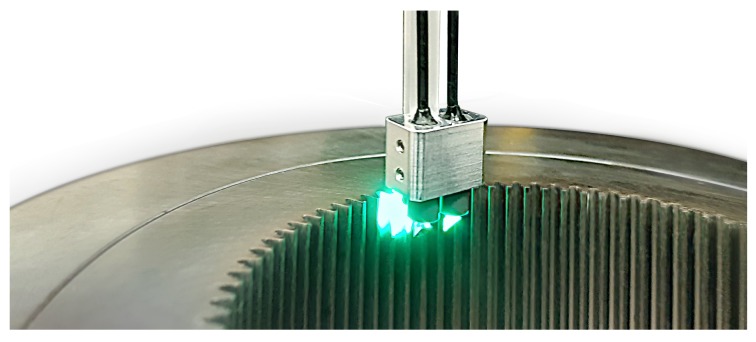
Specimen and measuring head while projecting structured light.

The measuring system was developed within the last years and is intended to perform measurements in geometries which would be unreachable for most common systems. This is done by using a small and flexible measuring head. The system is based on the fringe projection approach in order to quantify the condition of sheet-bulk metal forming tools in an industrial environment [7]. An endoscopic sensor is needed to enable measurements while the tool remains inside the forming plant. Based on the maximum available space (∼10 cm high), the sensor features a small measuring head being mounted onto a robot guided arm. An overview of a planned application is given in Figure 1, showing a possible measurement inside a forming plant. The general objective of this setup is to enable in situ measurements of certain highly stressed features between a certain number of forming cycles. This ensures continuous monitoring of the forming tool without stopping the process or partially disassemble the forming tool. Measurement times of less than 10 s are therefore the aim of current research. This includes the positioning of the measuring head. The system shall offer inspections for a wide range of different specimens, including flat gearing geometries as shown in Figure 1 or more complex geometries with multiple inner features (as shown in Figure 2) which are hard to access for most measurement approaches.

Since the in situ determination of deviations and the quantification of wear is limited to certain single features, representing the condition of the entire specimen, it is further necessary to precisely measure the entire geometry of each forming tool enabling comprehensive analysis. Due to the given geometrical constraints and the limited bending radii (∼20 cm) of the fibers, the workspace of the kinematics robot is restricted, leading to an offline inspection setup which shall be presented here. To guarantee comparable results for both principle tasks, it is important to use the same measurement system with identical optics.

Based on this first industrial application, introducing a robust system for time-effective and automated full-field 3D measurements at high resolution, a multitude of possible applications and future research is conceivable. The given setup is expected to enable a much better characterization of abrasion and wear, leading to deeper process understanding and helps to optimize certain process parameters. Additionally, it provides the basis for in situ inspections by identifying highly stressed geometries.

## 3. Problem and Approach

**Figure 3:**
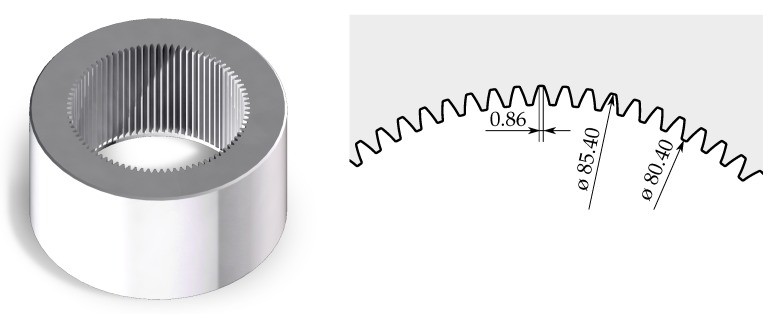
Overview and geometrical features of the specimen.

The approach is developed for measuring a specimen with multiple inside-lying geometries. Such a specimen is illustrated in Figure 3 and serves as a prerequisite for further investigations. The specimen features a height of 66 mm, an average diameter of 82.9 mm, 84 involute teeth and has a mass of ∼10 kg. Since most of the specimen’s features are hard to access for most other measurement approaches, the usage of the endoscopic fringe projection technique seems reasonable. Furthermore, the specimen could be utilized for possible future in situ inspections (mentioned in Section 2) of single features.

By combining several hundred measurements from different poses, the approach given here is trying to obtain detailed point cloud data and could therefore potentially measure all features. Since the whole inspection process shall be automated and less time consuming, a multi-axis kinematic is appropriate for traveling to all desired poses. A particular focus is therefore given to the alignment of all measurements. Given the fact of heavy, unwieldy specimens, various challenges affect precise alignment based on positioning data. This is especially relevant when the rotational axes are slightly misaligned. Moreover, the kinematics and the hand-eye transformation remain uncalibrated in order to meet the requirements of rapid inspection.

**Figure 4:**
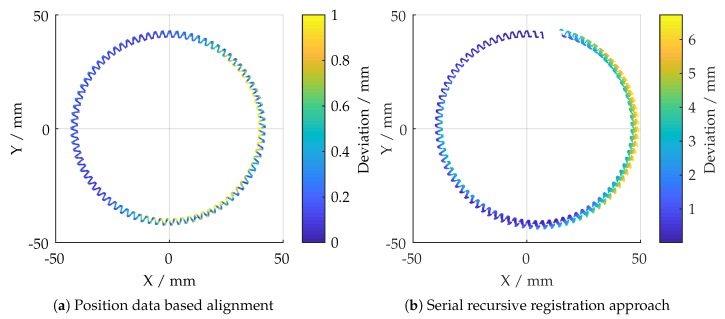
Comparison of different alignment approaches.

Figure 4a shows the deviations by aligning all measurements based on the positioning data only. It can be observed that some areas are affected by systematic misalignment, leading to meaningless results. The calculated deviation is based on an Euclidean metric, given in Section 7.4. It would, therefore, be appropriate to use a registration algorithm in order to optimize the overall alignment. A key challenge for stitching large datasets is to ensure that recursive serial registration is robust against drifting effects, caused by an accumulation of uncorrected errors. Despite featuring big overlapping areas in the to be registered point clouds, a conventional registration approach has not performed well. An example is given in Figure 4b. With an almost ideal set of starting values, the algorithm attempted to register all radial datasets in an anti-clockwise direction, leading to an accumulated error.

The experimental setup is designed to analyze the condition of sheet metal forming tools after certain cycles. The specimens are machined with high precision and reference geometry data is available. Therefore, the assumption was made that form deviations are negligibly small and computer-aided design (CAD) data based registration can be used to align the measured data. While this should allow a detailed examination of local features like gear geometries, it may reduce sensitivity to detect global deviations. The following sections aim at giving an overview of all crucial data processing algorithms and show first results.

## 4. Experimental Setup

**Figure 5:**
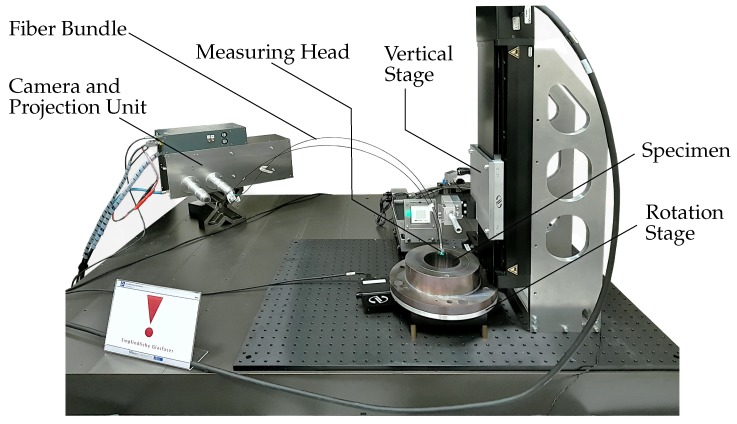
Experimental setup with major components.

In Figure 5 an overview of the experimental setup is given. The specimen is mounted onto a URS150BCC rotation stage, supplied by Newport Corporation (Irvine, CA, USA) with a typical accuracy of ±15 mdeg. The measuring head is mounted onto a M-IMS300V motorized vertical stage with a typical accuracy of ±5 µm which is also supplied by Newport Corporation. To measure the entire specimen, 588 separate measurements were performed. This includes 84 steps in radial and 7 steps in the axial direction (7 × 84 = 588). The entire testing procedure lasts a maximum of 60 min (not including data fusion).

## 5. Hardware Components

**Figure 6:**
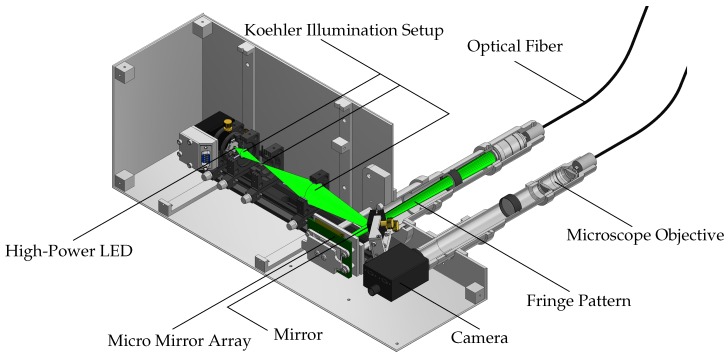
Overview of all major components of the light forming, fringe forming and camera unit.

Figure 6 shows the major components of the camera and projector unit. Polychromatic, Gaussian distributed green light with a peak wavelength of 528 nm or 505 nm, depending on the diode in use, is emitted by a high power LED (OSRAM Licht AG, Munich, Germany). A Koehler Illumination setup is used to create a homogeneous spot which is imaged (using an additional mirror) onto a digital micro mirror device (DMD) by Texas Instruments (Dallas, TX, USA) with 1024 × 768 individual adjustable mirrors. The DMD is used to create the projected patterns. Previous beam-shaping setups utilized a laser beam which was homogenized by a rotating diffusor and two microlens arrays (fly eye). Unfortunately, this approach led to higher measurement uncertainties, mainly related to speckle interference [8].

The fringes, created by the DMD, are injected into an endoscopic fiber by the use of a microscope objective and a tube lens. The fiber itself is a combination of multiple flexible glass fiber cores, combined to a fiber bundle in a common cladding. The fiber in use features 100,000 individual fiber cores (pixels) at a diameter of 1.7 mm and a length of 1 m and is supplied by Fujikura Ltd. (Tokio, Japan). The fiber is connected to a measuring head where the pattern is projected onto the specimen by using a gradient-index (GRIN) rod lens (Grintech GmbH, Jena, Germany). Due to the small apertures of the fiber, the light source has to feature a comparatively small chip diameter, in order to reduce the loss of light. The maximum optical power output (white projector image), measured in focus, is approximately 5.8 mW [8]. The projection is observed by a Point Grey GS3-U3-23S6M-C industrial CMOS camera (FLIR Integrated Imaging Solutions GmbH, Ludwigsburg, Germany) through another identical fiber and GRIN rod lens. Since the projection is green, an additional optical bandpass filter is provided.

Depending on the reflectance characteristics of the specimen, typical exposure times range from 10 to 50 ms. Since technical surface geometries exhibit highly varying reflectivity, the dynamic range of the sensor can be enhanced by combining differently exposed images to a high dynamic range image. Usually, eight different projection patterns are required to perform a measurement.

**Figure 7:**
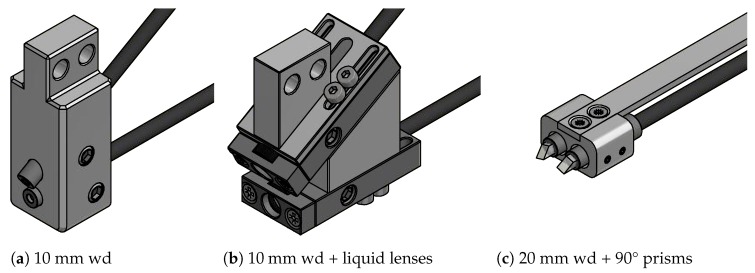
Overview of the measuring heads currently in use (scale 1:1).

Different measuring head configurations have emerged throughout the development, each optimized for a certain set of requirements. This section provides an overview of the different types and shall outline each individual scope of application. Figure 7a shows a measuring head with a 10 mm working distance (wd). Between the two pairs of fibers and optics, a 30° triangulation base is formed. The measuring volume is approximately 6 mm × 6 mm × 3 mm [9]. This measuring head features the lowest measurement uncertainty and the highest magnification alike. Figure 7c shows the design, using gradient-index rod lenses with 20 mm working distance, combined with additional mirror prisms. Therefore, a more compact design is realized, allowing a parallel arrangement of the fibers. The measuring volume is approximately 10 mm × 10 mm × 4 mm [9]. To enhance the depth of field of the measuring head with 10 mm working distance, additional liquid lenses can be added, which lead to the design of Figure 7b.

The measuring head, using two liquid lenses, offers a good compromise between maximum focus and depth of field. Since two liquid lenses add ten more optic interfaces to the optical system, a loss of approximately 50 to 55% optical power was measured in focus. Other drawbacks include the need for a time-effective autofocus, the electrical control setup, the much more extensive calibration and the larger physical size of the measuring head itself.

The specimen requires a depth of field of approximately 2.5 mm (compare Figure 7c) which is close to the limit of the 10 mm GRIN rod lens measuring head. Because of the parallel and rotated arrangement of the fibers, the only measuring head allowing movement into the specimen is the one using the design in Figure 7c. Because of the larger working distance and lower magnification, a bigger measuring field also reduces the required number of measurements to acquire the entire shape of the specimen but also leading to higher measurement uncertainties.

## 6. System Capabilities and Limitations

To quantify the accuracy of a single measurement, several external factors affecting the results should be taken into account:

- Optical properties of the technical surface
- Working distance, field curvature and depth of field (DOF) of the optics
- Size and form of the specimen
- Orientation between measuring head and the specimen
- Stray light and light scattering

The evident limitation of triangulation-based optical measurement approaches is given by the optical properties of the specimen. If the optical beam path is not unambiguously reconstructable, a measurement cannot be performed. This applies particularly to (partially) transparent surfaces. However, specular reflection can also be considered as a problem. By the use of HDR-imaging, a measurement can be performed in the highlight spot as long as the specimen does not feature structures causing multi reflections. Particularly due to the involute tooth flanks in combination with the fine surface properties of the specimen, it would be impossible to triangulate useful results. To overcome this, anti-glossy spray is applied. This leads to changes of the geometric form of the specimen and shall later be evaluated.

As mentioned in Section 5, all measuring heads feature different focus ranges. Those are also highly correlated to the radial position in the field of view due to field curvature. Since the 10 mm GRIN rod lens features the highest magnification, the best results can be produced. However, an off-focus orientation or different lens setups can also reduce the measurement uncertainty since noise or other artifacts are blurred. This is strongly dependent upon the spatial characteristics of the specimen. Other factors, regarding the form of the specimen, are connected to the geometrical orientation of each surface and the fringe frequency. If fringes are projected at an acute angle to a surface (like the lateral gear surfaces), aliasing effects occur due to the finite number of camera pixels and fiber bundles and can produce noise, reducing the accuracy. To overcome aliasing effects, the generation of adaptive projection patterns based on local frequency modulated fringes [10] is supposed to be implemented in the future.

## 7. Data Processing

**Figure 8:**
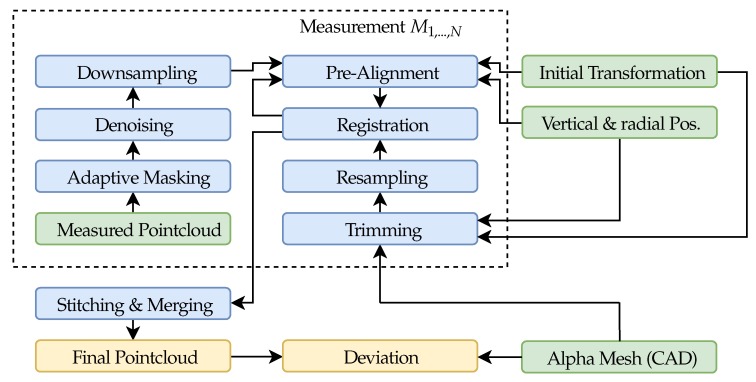
Flowchart of all significant data processing operations (green: input, blue: data processing, orange: output).

The flowchart, given in Figure 8, provides an overview of all critical data processing algorithms. The routine can later be parallel processed, especially against the background of reducing data volume. Currently, 80 GB of data must be cached and then processed.

At first, a rough initial transformation $T_{\mathrm{initial}}$ is performed to help the registration algorithm converging by aligning the first measurement with the CAD-originated shape data, from now on named alpha point data. A detailed explanation of $T_{\mathrm{initial}}$ is given in Section 7.1.

The alpha coordinate system is placed at the center of rotation. By using the radial position data of the stage $\phi_{\mathrm{curr}}$, any additional measured point cloud can be roughly pre-aligned by the use of a rigid body transformation $T_{\mathrm{curr}}$ according to Equation (1). Vertical positioning is considered by shifting the data along the rotation axis.

(1) $$T_{\mathrm{curr}}=T_{\mathrm{initial}}\cdot \left[\begin{array}{cccc} cos(\phi_{\mathrm{curr}}) & -sin(\phi_{\mathrm{curr}}) & 0 & 0\\ sin(\phi_{\mathrm{curr}}) & cos(\phi_{\mathrm{curr}}) & 0 & 0\\ 0 & 0 & 1 & z_{\mathrm{curr}}\\ 0 & 0 & 0 & 1 \end{array}\right]$$

Before starting the registration operation, a denoising algorithm is applied, removing separately scattered points. The algorithm is based on the work of [11] and computes the distribution of point neighbors distances. The points with mean distances outside a threshold are trimmed from the data. To further improve convergence and to speed up calculations, a random downsampling is useful and is set to 10% of the original samples (leading to ∼100,000 samples in each dataset).

After pre-aligning, the denoised and downsampled measured data is registered to the alpha data. To enhance performance, only features which exist in the measured point cloud shall be taken into account. The current transformation $T_{\mathrm{curr}}$ is therefore used to trim the alpha data based on an region of interest (ROI) which originates from the center of mass (see Equation (3)) of the pre-aligned data. The size of the ROI was determined to 10 mm in axial and 0.2 rad in radial direction. Further, as discussed in Section 7.3, the alpha data is then randomly resampled in order to obtain a point cloud, to which the measured data is registered, according to Section 7.4. The amount of created alpha samples is comparable to the number of samples in the measured point cloud.

After having aligned the first measurement with the alpha point cloud, the progress is repeated, starting with trimming and resampling of the next alpha geometries, based on the current rigid body transformation $T_{\mathrm{curr}}$. In this example, a single alpha template could be used for all registration operations. To ensure that this registration approach is robust against misalignment of the rotational axes or minor movements of the measuring head during the measurement, the initial transformation is continuously updated, based on the last registration operation. This could potentially lead to error drifting by an accumulation of minor errors during registration. A possible reason could be distortion which has not perfectly been removed due to calibration. To suppress this, big overlapping areas are featured in the to be registered point clouds. Another potential source of drifting effects is given by one remaining degree of freedom in each registration step. This is caused by the extruded shape of the specimen in the axial direction. As shown in Equation (2), the current registered point cloud is corrected by shifting the axial center of gravity to the intended position $z_{\mathrm{ref}}$ (based on the position data of the vertical stage).

(2) $$pc_{\mathrm{shift}}=\underset{\mathrm{Point}\;\mathrm{Cloud}}{\underset{︸}{\left[\begin{array}{ccc} x_{1} & y_{1} & z_{1}\\ \vdots & \vdots & \vdots \\ x_{n} & y_{n} & z_{n} \end{array}\right]}}+\underset{Z-\mathrm{Shift}}{\underset{︸}{\left[\begin{array}{ccc} 0 & 0 & \left(z_{\mathrm{ref}}-\frac{1}{n}\sum_{i=1}^{n}z_{i}\right) \end{array}\right]}}$$

The process of stitching and merging combines two registered point clouds with each other by removing points, mainly in the overlapping area. This procedure is repeated for all data sets. Currently, this is done by using the Computer Vision System Toolbox which is part of Matlab, supplied by The MathWorks Inc. (Natick, MA, USA).

**Figure 9:**
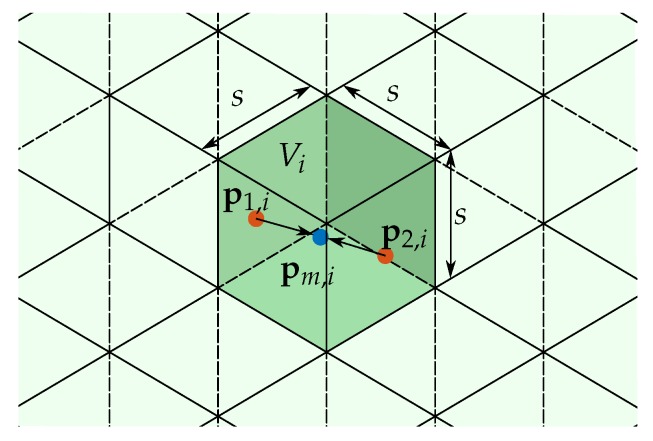
Process of merging points inside one voxel.

The process of merging is illustrated in Figure 9. For each voxel $V_{i}\in \bar{V}$, where $\bar{V}$ is the volume in which all points are scattered, all points within $V_{i}$ are merged to one single point $p_{m,i}\in V_{i}$ according to the center of mass (see Equation (3)). The size of each voxel is determined by the grid step size *s*.

The final deviation map is then calculated, based on the distance metric presented in Section 7.4 which was already used in each registration operation.

### 7.1. Initial Transformation

**Figure 10:**
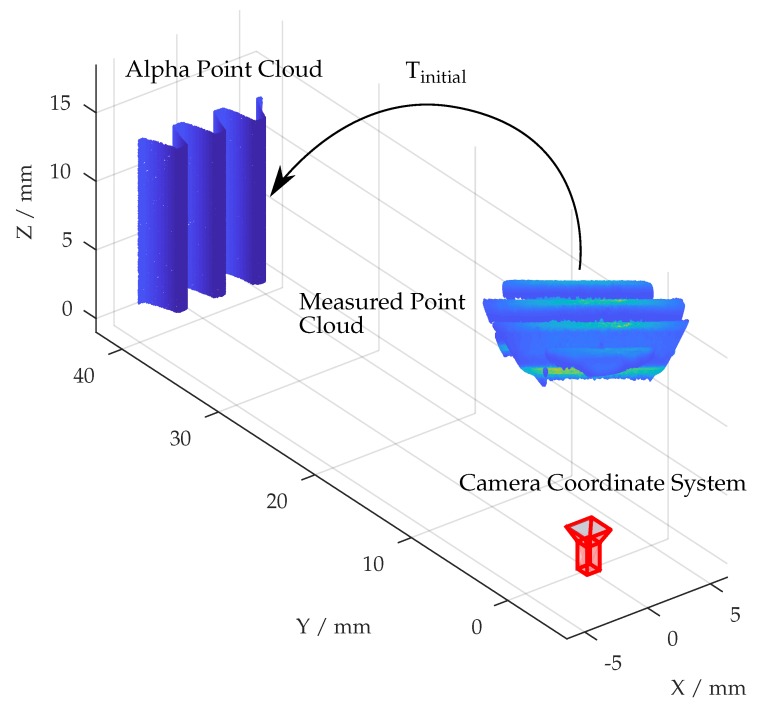
Initial transformation from camera coordinate system with respect to the alpha point cloud.

The determination of the initial transformation is the only operation requiring user input. By selecting corresponding point sets *P* and *X* from both point clouds, a rigid transformation is computed. The goal behind this is, as shown in Figure 10, to transform each following measurement as good as possible from the camera coordinate system into the alpha coordinate system, which is given by CAD data.

The algorithms are based on the work of [12] and [13], starting by calculating the center of mass of all point sets *P* and *X* according to Equation (3).

(3) $$\mu_{p}=\frac{1}{n_{p}}\sum_{i=1}^{n_{p}}p_{i}\;\mathrm{and}\;\mu_{x}=\frac{1}{n_{x}}\sum_{i=1}^{n_{x}}x_{i}$$

The square cross-covariance matrix $\Sigma_{\mathrm{px}}$ is given according to Equation (4) [12] and is used to find the optimum rotation through computing singular value decomposition: $\Sigma_{\mathrm{px}}=U\Sigma V^{T}$. The resulting rigid body transformation is given in Equation (5). It should be mentioned that this approach can compute results in which the determinant of the rotation matrix *R* is less than zero. In this special reflection case, the third column of *R* should be multiplied by −1.

(4) $$\Sigma_{\mathrm{px}}=\frac{1}{n_{p}}\sum_{i=1}^{n_{p}}\left[\left(p_{i}-\mu_{p}\right){\left(x_{i}-\mu_{x}\right)}^{T}\right]=\frac{1}{n_{p}}\sum_{i=1}^{n_{p}}\left[p_{i}x_{i}^{T}\right]-\mu_{p}\mu_{x}^{T}$$

(5) $$T_{\mathrm{initial}}=\left[\begin{array}{cc} \overset{R}{\overset{︷}{VU^{T}}} & \overset{t}{\overset{︷}{\mu_{x}-VU^{T}\mu_{p}}}\\ 0^{T} & 1 \end{array}\right]$$

### 7.2. Outlier Removal

To improve the quality of the measurement and to remove non-plausible data, each point cloud is masked. Since each point of the point cloud stems from one pixel in the camera coordinate system, the use of a two-dimensional mask can be applied to the measured point cloud. A manually defined region of interest is not useful for very large datasets. In addition, a fixed mask is not robust against misalignments since features are consequently not always at a fixed position in the camera coordinate system. Due to a misalignment of the rotation axes of the stage and the specimen, this could lead to inaccurately trimmed data. Therefore, the camera data is used to quantify the condition of each pixel and it’s corresponding triangulated point based on two-dimensional signal characteristics. The following approach has proven to reduce the measurement uncertainty by eliminating outlier points where triangulation did not work as well.

**Figure 11:**
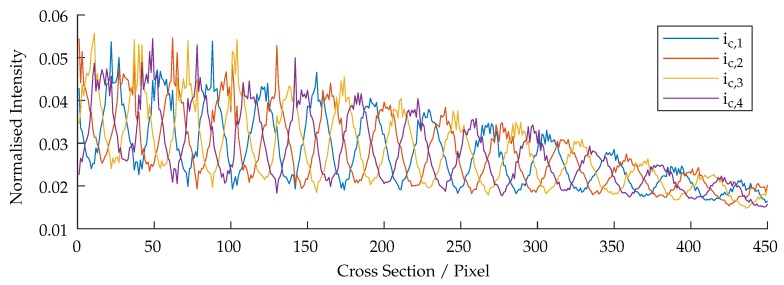
Cross-section of all captured images with the phase-shift of the highest frequency.

**Figure 12:**
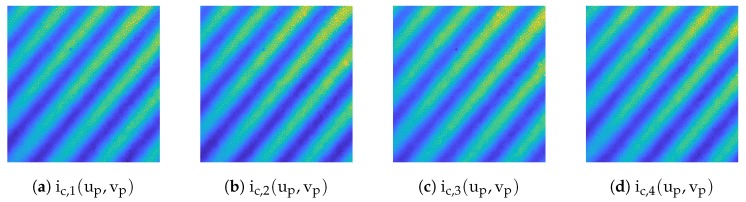
Cropped camera images showing shifted fringes of the highest frequency being projected onto a plane.

**Figure 13:**
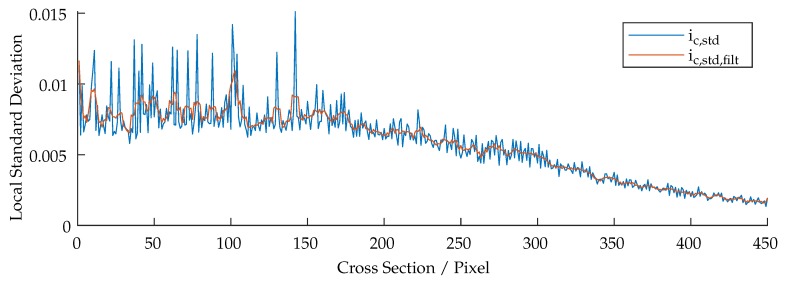
Calculated and filtered local standard deviation of the cross sections of Figure 11.

The presented system is based on the fringe projection approach (active stereo vision). The correspondence between camera and projector is determined by sinusoidal patterns being shifted through the scene. Figure 11 shows a cross-section, (perpendicular to the wavefronts) of all projected and phase shifted patterns of one frequency which are shown partially in Figure 12. The patterns were projected onto a diffuse plane. It can be observed, that the projected patterns are in focus within the range of 0–150 pixels, resulting in high amplitude oscillations. The cross-section also shows areas of bad illumination. By assuming that a high pixel-wise deviation within all shifted patterns of one frequency is a suitable criteria of validating the quality of each triangulated point, this approach can be adopted by calculating the local standard deviation per pixel through all phase shifts of each frequency which is shown in Equation (6), where $i_{c,\mu }\left(u_{p},v_{p}\right)$ is the mean value of each pixel. The result is shown in Figure 13.

(6) $$i_{c,\mathrm{std}}\left(u_{p},v_{p}\right)=\sqrt{\frac{1}{n}{\left(\sum_{i=1}^{n}i_{c,i}\left(u_{p},v_{p}\right)-i_{c,\mu }\left(u_{p},v_{p}\right)\right)}^{2}}$$

**Figure 14:**
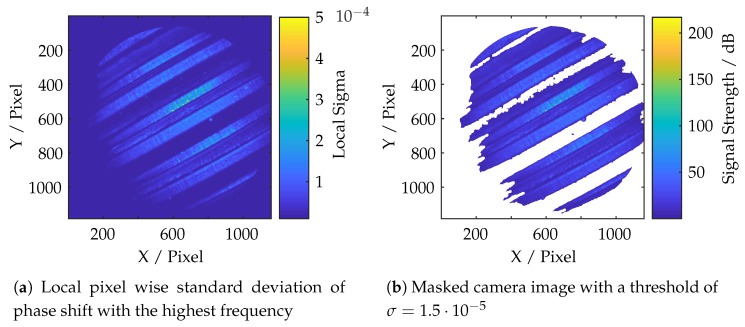
Overview of signal based masking in camera coordinate space.

As can be seen in Figure 11, Figure 12 and Figure 13, the oscillations carry additional high frequency noise when in focus. This effect is intensified by sampling the image by the use of fiber cores. Therefore, additional smoothing filters, based on a Gaussian approximation, are applied to $i_{c,\mathrm{std}}\left(u_{p},v_{p}\right)$. Finally, the data is thresholded and applied to each point cloud. Figure 14 shows the masking process. The threshold in use is rather less aggressive. However, as can be seen in Figure 14b, some lateral geometries have already been deleted. This is presumably due to the arrangement of the two fibers and the optics, because the projection comes from a certain lateral direction and so some structures remain badly illuminated due to shadowing.

By merging all measured point clouds, captured from different perspectives, bigger holes in certain point clouds can be filled. The trimming threshold influences the results and conclusions of the inspection process. A more aggressive threshold leads to a decrease of the measurement uncertainty while reducing data density at areas of bad illumination. The determination of the threshold value should therefore always be done in accordance with the individual geometrical characteristics and possible wear of each specimen.

### 7.3. Resampling the Alpha Point Cloud

In order to calculate a deviation and to apply a registration algorithm, an alpha point cloud must be created from a common CAD file. The algorithms are optimized for the .ply file format, which stores the information in the face-vertex format. This is an object representation by listing all vertices, forming a face (face list) and listing all faces; each vertex is referring to (vertex list).

**Figure 15:**
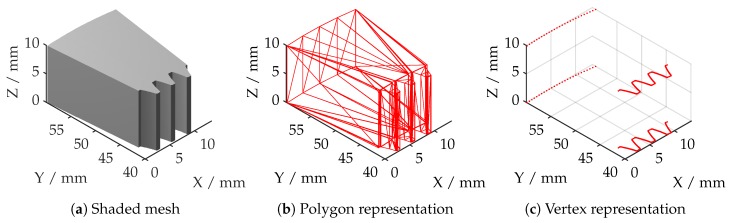
The stored information in computer-aided design (CAD) reference geometry.

In order to enhance performance and to ensure that the registration algorithm guarantees comparable results for all given measurements with the same set of parameters (see Table 1), the reference alpha point cloud should match the shape of the measured point cloud as well as possible. Therefore, the alpha mesh is sliced into smaller geometries with the rough size of the measurement volume of the measuring head in use. Figure 15a shows an extracted part of the alpha geometry. Figure 15b,c show the stored polygonial and vertex data. In this case, the entire structure is periodical in the radial and axial direction, so the sliced template could be used for all registration operations. The trimming of a template out of the whole reference mesh is automated by using the initial transformation and the position data of the cinematics, each measurement is corresponding to.

The first step is to trim all vertices and corresponding faces beyond the gear geometries. This is done by applying a radial threshold (50 mm) to the data. To create additional point cloud data, points on each polygon have to be sampled. The total number of sampling points for each polygon is calculated by comparing the area of each polygon with the total area of all polygons and the number of required samples. Since these polygons are triangles, the surface area in 3D space is given by the half cross product formula.

A method for generating unbiased random points with respect to the surface area was introduced by [14] and has been adopted. For a given triangle (with vertices A,B,C) a point on its surface can randomly be constructed by the following equation:

(7) $$P=\left(1-\sqrt{r_{1}}\right)A+\sqrt{r_{1}}\left(1-r_{2}\right)B+\sqrt{r_{1}}r_{2}C$$

By generating the two random numbers $r_{1}$ and $r_{2}$ and by taking the square root of $r_{1}$, a random point with respect to the surface area is constructed. $r_{1}$ sets the percentage from vertex *A* to the opposing edge $\bar{BC}$, leading to another edge on which $r_{2}$ defines the final point *P*. The sampling of 100,000 points lasted for about half a second. It has been experimentally proven to be important to generate uniformly distributed random numbers. Otherwise, this approach does not produce useful results and sampled points seem to accumulate in one edge of each triangle.

### 7.4. Distance Metrics and Registration

By the use of a proper distance metric, a point to point distance between two point clouds can be calculated. The Minkowksi distance of order *p* between two point clouds $X=(x_{1},\ldots ,x_{n})$ and $Y=(y_{1},\ldots ,y_{m})$ is defined as:

(8) $$d_{\mathrm{mk}}=\sqrt[{p}]{\sum_{i=1}^{n}{\left|x_{i}-y_{i}\right|}^{p}}$$

where *p* is ranging from 1 (City block distance) to *∞* (Chebychev distance). For p=2, the Minkowski distance gives the Euclidean distance, for each pair of points. To get information about the deviation of the measured point cloud to the alpha point cloud, the smallest distance in $d_{\mathrm{mk}}\in R^{nxm}$ is obtained by sorting the distances in each column in ascending order (set of closest points) or extracting the smallest distance. As the data sets can be large, this calculation is time intense (∼ 30 min for all stitched point clouds), despite using GPU computation.

The metric is also used for the registration of each point cloud by combining the approach based on the Euclidean distance with a rigid affine transformation to define the mean-squares objective function:

(9) $$F\left(R,t\right)=\frac{1}{N_{p}}\sum_{i=1}^{n}\left|\right|x_{i}-R\cdot y_{i}-t{\left|\right|}^{2}$$

Let $N_{p}$ be the number of pairs, representing correspondences and *R* the rotation matrix and t the translation vector of the unknown affine transformation. The goal is to find the least squares rotation and translation by using any optimization method, such as steepest descent, conjugate gradient or simplex [15]. More recent approaches aim to reduce computation time by using the much faster quaternion method, introduced by [16,17].

In this case, the iterative closest points (ICP) algorithm from the computer vision system toolbox (mentioned in Section 7) is used for each registration operation. The main parameters are listed in Table 1. Based on the given inlier ratio, specifying the percentage of matched points considered as inliers, the algorithm is estimating the optimal rigid body transformation between both sets of points.

**Figure 16:**
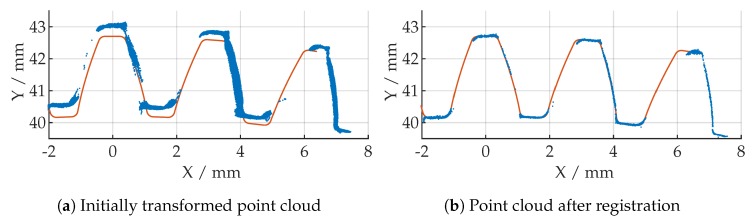
Result of registration operation (blue: measured point cloud, orange: alpha point cloud).

**Figure 17:**
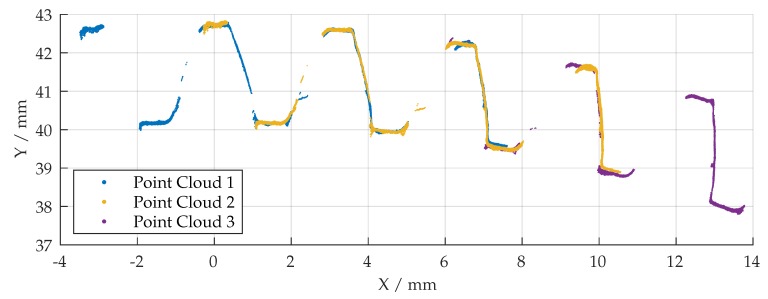
Result of registration approach for the first three point clouds.

While the total number of iterations in each registration operation is limited to 25, the tolerance of absolute difference between consecutive ICP iterations (in translation and rotation) provides an additional stop criterion. In combination with good initial values, the algorithm aborts the calculation based on these termination criteria in most cases. A typical registration operation takes <10 s by calculating 5 iterations on the average. Figure 16 shows a registration operation, where the initially transformed point cloud is fitted, based on the given termination criteria, into the alpha data to calculate a possible deviation later. The results for the first three measurements are shown in Figure 17. The process is repeated for all measured point clouds and the initial transformation matrix is updated after each registration operation.

## 8. Results

**Figure 18:**
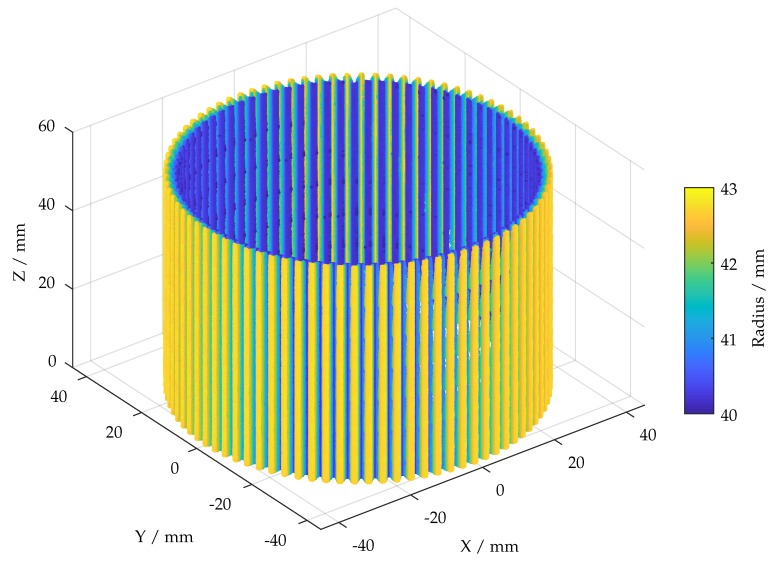
Final point cloud, according to flowchart Figure 8 (downsampled to 10 % of the actual size).

Figure 18 shows the final point cloud which was created according to the flowchart which is given in Figure 8. Each of the measured 588 point clouds was masked, based on the pixel-wise filtered standard deviation of the phase shift with the highest frequency, denoised and then downsampled to 10 % of the original size. By the use of an initial transformation and the positioning data of the stages, a registration algorithm was able to align each of the measurements by using an alpha point cloud which was resampled from trimmed CAD data. Finally, the data was merged, leading to ∼42.1 mio. points. It is noticeable that the results are still fragmentary and gaps are mainly found at the side edges and back edges (originating from the rotation center). This is not involving the front surface on which the major influence of abrasion is expected. The point cloud can be loaded into common CAD software to supply actual geometry data. In order to secure wide compatibility, additional meshing might be required.

**Figure 19:**
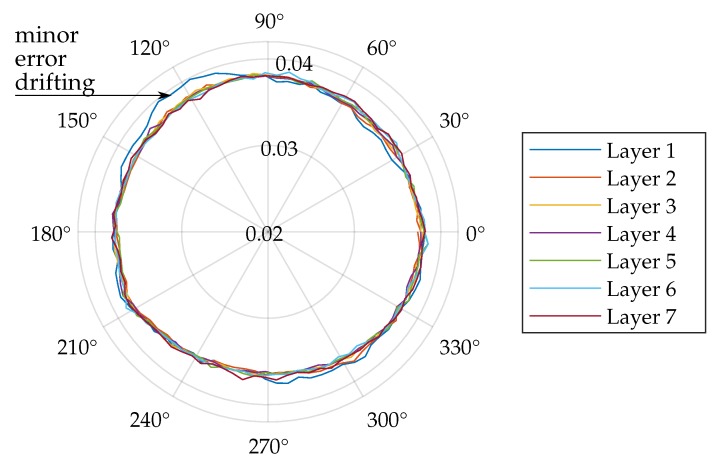
Root mean square (RMS) error of all registration operations with respect to each vertical layer and radial position

Figure 19 shows the remaining root mean square (RMS) error with respect to the Euclidean metric for all 588 registration operations after the ICP stopped due to its termination criterion (see Section 7.4). The layers correspond to the axial position of the stage. It can be assumed that the first layer is affected by minor drifting effects during registration. Shape deviations can presumably be excluded since all other layers seem to be unaffected. Uniform drifting effects concerning several layers could indicate significant shape deviations. The condition of certain features could still be quantified but the reconstruction of the whole shape would not be appropriate in this case with the given registration approach.

**Figure 20:**
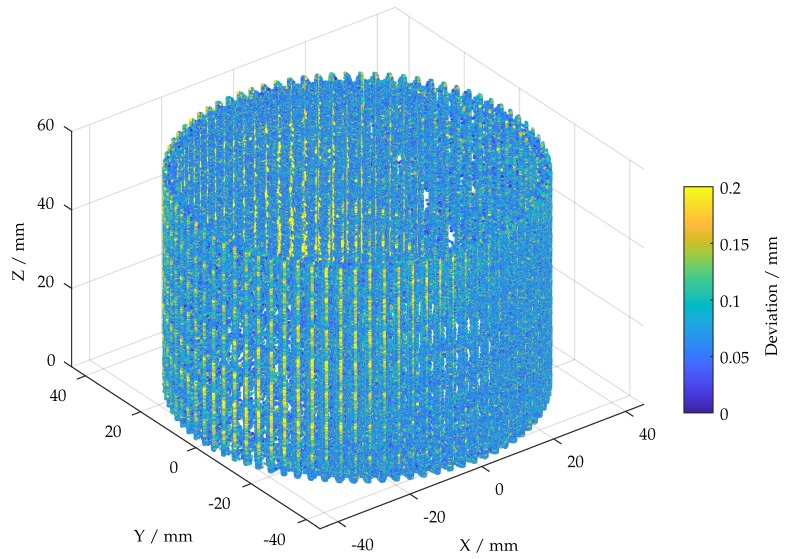
Calculated deviations projected onto a cylinder.

In order to make assessments with regard to indications of possible defects, the point cloud from Figure 18 is used to calculate the overall point-wise deviation based on the distance metric and alpha point cloud from Section 7. Despite using GPU acceleration, this last calculation is time-consuming (∼30 min). This is due to large datasets. The calculated deviations are color-coded in Figure 20. The major deviations range between 0 and 0.2 mm. Noticeable deviations are mainly found at some back edges, presumably caused due to poor illumination and accumulations of anti-glossy spray. Except that, deviations are spread randomly throughout the entire point cloud. This result is supposed to provide comprehensive data to support maintenance. Furthermore, highly stressed features can be identified in order to enable possible future in situ inspection (introduced in Section 2).

**Figure 21:**
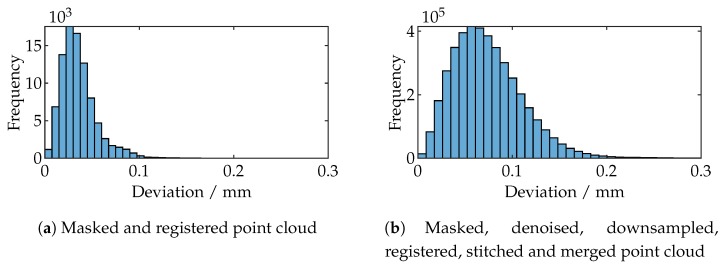
Comparison of the deviation histograms of a single measurement and the final point cloud of all 588 measurements.

Figure 21b shows the histogram of all deviations. The arithmetic average is 0.0741 mm with a standard deviation of 0.0371 mm. In comparison to this, Figure 21a shows the histogram of one single measurement.

The arithmetic average is 0.0356 mm with a standard deviation of 0.0188 mm. The greater measurement uncertainty is presumably caused by averaging (due to merging) all minor misalignments throughout all 588 measurements.

## 9. Discussion

A robust system for time-effective and automated full-field 3D measurements at high resolution for industrial applications has been introduced. The compact and flexible design of the 3D measuring endoscope, based on the endoscopic fringe projection approach, can be used to measure the shape of geometries which remain out of reach for traditional tactile or other measurement principles. In this application, several hundred single measurements were performed to scan all features of a rotationally symmetric forming tool. Much better stitching can be achieved by using a CAD based registration approach. This is especially relevant when the rotational axes are slightly misaligned and the kinematics and the hand-eye transformation remain uncalibrated and therefore the positing data of the stages cannot be used to precisely stitch all point clouds. Against the background of rapid inspection and heavy and unwieldy specimen, requiring extremely precise alignment, this approach can be considered reasonable. By utilizing and resampling CAD data, the registration can successfully stitch all measured data and calculate the overall deviation. The registration approach shifts the focus in favor of a very detailed examination of local features like gear geometries while reducing the sensitivity to detect shape deviations which should be taken into consideration.

To reduce fragmentation at the back edges, the number of vertical measuring positions should be enlarged. The fragmentation at the side edges is probably caused by the arrangement of the camera and projector optics at the measuring head. A clear improvement could be achieved by adding another projector into the system, arranged on the other side of the camera.

Furthermore, this setup is supposed to enable a much better characterization of abrasion and wear. This can be used to optimize the threshold level, introduced in Section 7.2, setting the ratio between measurement density and measurement uncertainty. This ensures that no defects are skipped while the system is sufficiently sensitive to detect all possible types of defects. Beyond that, the deeper process of understanding can be used to optimize certain process parameters.

Since the original geometrical properties of the specimen are not known exactly, the measurement uncertainty cannot be concluded. A potentially significant source of deviation is given by the layer of applied anti-glossy spray which presumably leads to a more or less constant deviation offset. Nevertheless, despite this first approach, it is expected that future potential damage of the specimen can be identified and measured reliably. A detailed assessment of the systems performance and sensitivity to identify various defects should be made shortly after enough data has been collected and the system has proven to be is suitable for permanent use in various industrial applications.

## 10. Future Improvements

This initial approach is providing scope for further improvements. It is supposed, that higher uncertainty in contrast to one ideal single measurement appears to be connected to the registration and the merging operation. Whereas the error susceptibility of the registration seems difficult to reduce, merging could be significantly improved. Instead of combining the points in each voxel the filter should keep only the points of good condition in future developments. Therefore, the same map which was originally used to mask each point cloud (see Section 7.2) could be used. Furthermore, the local quality of calibration could be a considerable factor since distortion is a major error source at the edges of the camera image. A selection could be done by comparing the estimated local reprojection error of all points within one voxel. The reprojection error is an important criterion to estimate the calibration quality for each calibrated feature, based on the camera calibration by [18,19].

Measuring and all data processing took about 3 h, which can be significantly accelerated. At first by parallelizing point cloud computation and measurement. Despite GPU support, the most time-consuming step is calculating the final point-wise deviation based on the smallest Euclidean distance of the alpha and measured point cloud. To increase performance, additional voxel filters shall be applied. An alternative approach would be the transformation of both point clouds into cylinder coordinates and the rearrangement of every axial layer in order of ascending angle. Each layer can then be resampled by interpolation according to a given grid. Finally, all points can be projected onto a plane. By subtracting both images (alpha data and measured data), the deviation can be calculated. According to a first assessment, a deviation can be calculated within seconds while harboring minor interpolation errors.

A more precise approach would be the calculation of the deviation based on a point-plane metric by using the polygon data stored in the alpha mesh. This metric could also be used for the registration algorithm superseding the alpha point cloud resampling step. 

## Figures and Tables

**Table 1:** Parameterisation of the iterative closest points (ICP) registration algorithm.

Inlier Ratio		0.8
Maximum Number of Iterations		25
Tolerance	$R_{\mathrm{diff}}$	$0.005^{\circ }$
	$t_{\mathrm{diff}}$	0.001 mm

## Abbreviations

The following abbreviations are used in this manuscript:

CAD	Computer aided design
CMOS	Complementary metal-oxide-semiconductor
DOF	Depth of field
GPU	Graphics processing unit
GRIN	Gradient index
HDR	High dynamic range
ICP	Iterative closest point
LED	Light-emitting diode
RMS	Root mean square
WD	Working distance
